# Clinical Speech fMRI in Children and Adolescents: Development of an Optimal Protocol and Analysis Algorithm

**DOI:** 10.1007/s00062-021-01097-z

**Published:** 2021-10-06

**Authors:** Hannah Krafft, Martin Staudt

**Affiliations:** 1 Center for Pediatric Neurology, Neurorehabilitation and Epileptology, Schön Clinic Vogtareuth, Vogtareuth, Germany; 2 Department of Pediatric Neurology and Developmental Medicine, Children’s Hospital, University of Tübingen, Tübingen, Germany; 3 Department of Electrophysiology, German Heart Center Munich, Technical University of Munich, Munich, Germany

**Keywords:** Pediatric patients, Functional magnetic resonance imaging tasks, Language lateralization, Epilepsy surgery, Wada test

## Abstract

**Purpose:**

In patients with drug-resistant focal epilepsy, surgical resection is often the only treatment option to achieve long-term seizure control. Prior to brain surgery involving potential language areas, identification of hemispheric language dominance is crucial. Our group developed and validated a functional magnetic resonance imaging (fMRI) battery of four pediatric language tasks. The present study aimed at optimizing fMRI data acquisition and analysis using these tasks.

**Methods:**

We retrospectively analyzed speech fMRI examinations of 114 neuropediatric patients (age range 5.8–17.8 years) who were examined prior to possible epilepsy surgery. In order to evaluate hemispheric language dominance, 1–4 language tasks (vowel identification task VIT, word-chain task WCT, beep-story task BST, synonym task SYT) were measured.

**Results:**

Language dominance was classified using fMRI activation in the 13 validly lateralizing ROIs (VLR) in frontal, temporal and parietal lobes and cerebellum of the recent validation study from our group: 47/114 patients were classified as left-dominant, 34/114 as bilateral and 6/114 as right-dominant. In an attempt to enlarge the set of VLR, we then compared for each task agreement of these ROI activations with the classified language dominance. We found four additional task-specific ROIs showing concordant activation and activation in ≥ 10 sessions, which we termed validly lateralizing (VLR_new_). The new VLRs were: for VIT the temporal language area and for SYT the middle frontal gyrus, the intraparietal sulcus and cerebellum. Finally, in order to find the optimal sequence of measuring the different tasks, we analyzed the success rates of single tasks and all possible task combinations. The sequence 1) VIT 2) WCT 3) BST 4) SYT was identified as the optimal sequence, yielding the highest chance to obtain reliable results even when the fMRI examination has to be stopped, e.g., due to lack of cooperation.

**Conclusion:**

Our suggested task order together with the enlarged set of VLR_new_ may contribute to optimize pediatric speech fMRI in a clinical setting.

**Supplementary Information:**

The online version of this article (10.1007/s00062-021-01097-z) contains supplementary material, which is available to authorized users.

## Introduction

In patients with drug-resistant focal epilepsy, surgical resection is often the only treatment option to achieve lasting relief from seizures. Prior to brain surgery near language areas, identification of hemispheric language dominance is crucial [1–4]. In patients with neurologic disorders, such as epilepsy or structural brain lesions, particularly pediatric patients with early onset of disease, atypical language representation is up to 77% more frequent than in healthy subjects [2–5]. When such patients are evaluated for neurosurgical procedures involving language-relevant structures, the determination of hemispheric language dominance is crucial.

Traditionally, for this purpose the invasive Wada test [6] is applied: language tasks are performed during temporary anesthesia of one hemisphere by injection of sodium amobarbital into the internal carotid artery. Language dominance is unilateral when all language tasks are correctly performed with the patient under anesthesia in one hemisphere and inability to perform tasks under anesthesia of the contralateral hemisphere. Language is bilateral when inaccurate performance occurs following anesthesia of either hemisphere. The Wada test can still be regarded as the gold standard to determine hemispheric language dominance. Due to shortcomings, risks and side-effects of the Wada test, such as lack of a standard protocol, invasive catheterization of the carotid arteries, implying the risks of ischemic or hemorrhagic complications and exposure to ionizing radiation [7, 8], a noninvasive routine technique for evaluation of hemispheric language dominance is becoming increasingly more important [9].

Functional magnetic resonance imaging (fMRI) is reliably used for language lateralization (for reviews *see* [2, 9–12]) and provides detailed information on the localization of language areas in both hemispheres at once; however, caution is required for critical surgical decisions based on fMRI because activated areas might not necessarily be essential for language processing [13] or vice versa fMRI might miss critical language areas due to a lack of activation by the task [14].

In pediatric patients, fMRI faces methodological obstacles such as poorer compliance, increased motion artefacts and time spent in the scanner as limiting factors [5, 15–19]. The ideal fMRI language task paradigm needs to be appropriately challenging to produce reliable activation without overwhelming cognitively impaired or young children [20]. The tasks need to provide an appropriate balance of sensitivity and specificity for language-related activation and should provide reliable interhemispheric lateralization and intrahemispheric “localisation of language production and perception areas” [20].

For this purpose, our group has previously established an fMRI “task battery” to assess hemispheric language dominance in children as young as 6 years of age. The battery consists of four different tasks: the “vowel identification task” (VIT) [21], the “word-chain task” (WCT) [22], the “beep-story task” (BST) [23], and the “synonym task” (SYT) [21]. Using a “battery” of tasks is beneficial to target various language areas and display different linguistic components [24–28]. By combining more demanding language tasks (e.g., WCT and SYT) with easier tasks suitable for younger children (e.g., VIT) and with passive language tasks (e.g., BST), varying patient concentration and cooperation during the fMRI examination might be compensated [13]. This is especially relevant in the clinical examination of younger or cognitively impaired patients, when a fMRI examination often has to be stopped due to decline in motivation before all tasks can be measured. As with simple story listening, the BST requires low levels of cooperation and cognitive functioning and is therefore well-suited for younger or cognitively impaired children with difficulties performing an active task [23]. For the BST [23], however, cue words are replaced by sinusoidal tones, so that subjects can silently fill in the gaps, thus inducing active language processing in frontal areas during passive listening. For the control condition, subjects listen to a series of sinusoidal tones. For older children, capable of performing more demanding tasks, a simple story listening task might be too easy to achieve sufficient activation. Thus, active language production tasks are needed. For the WCT [22], patients are asked to produce silent chains of words, by thinking of a new word starting with the last letter of the previous word and so forth, thus facilitating a constant output of words during scanning. For the control condition, subjects are asked to rest. In the SYT [21] children decide if two visually presented words have the same meaning. For the control condition, a pair of nonsense letter strings are presented, and patients decide if they are identical or not. The VIT [21] is also a language decision task: patients identify a drawing (e.g., ship, ball), assign a name to it and then decide if the word contains the phoneme/i/(in German always spelled as “i”). For control, subjects are asked to analyze if a smaller puzzle piece is part of a larger picture of an abstract pattern or not. For VIT and SYT, the decision is communicated by pressing response buttons (yes/no) [21].

Furthermore, a decision about hemispheric language dominance is more reliable when based upon different tasks, especially in patients with bilateral language representation [29]. A combined task analysis increases the probability of discerning language-essential brain areas [29] and facilitates overall interpretation of laterality, since nearly all fMRI scans show some bilateral activation [30, 31].

In a recent fMRI validation study [1] 28 patients who underwent fMRI and the Wada test and/or experienced unchanged linguistic abilities after hemispherotomy were analyzed. The Wada test and unchanged language after hemispherotomy were used as the gold standard for validation. Using a region-specific evaluation of activation patterns, our group introduced, for each of these four tasks, task-specific regions of interest (ROIs) as valid for lateralization [1]. Thus, 13 valid, task-specific ROIs were identified, i.e., for WCT frontal operculum (FOP), inferior frontal gyrus (IFG), middle frontal gyrus (MFG), intraparietal sulcus (IPS) and cerebellum (CBM). For VIT the FOP, IFG and MFG were identified, for SYT, FOP, IFG and temporal language area (TLA), and for BST, IFG and MFG [1].

The purpose of the current study was to develop an optimized study protocol and evaluation strategy on the basis of this previous validation study [1], expanding the data set to a larger cohort of pediatric patients, also including patients with bilateral language representation, which had not been included in the validation study [1].

## Patients and Methods

### Patient Cohort

**Fig. 1:**
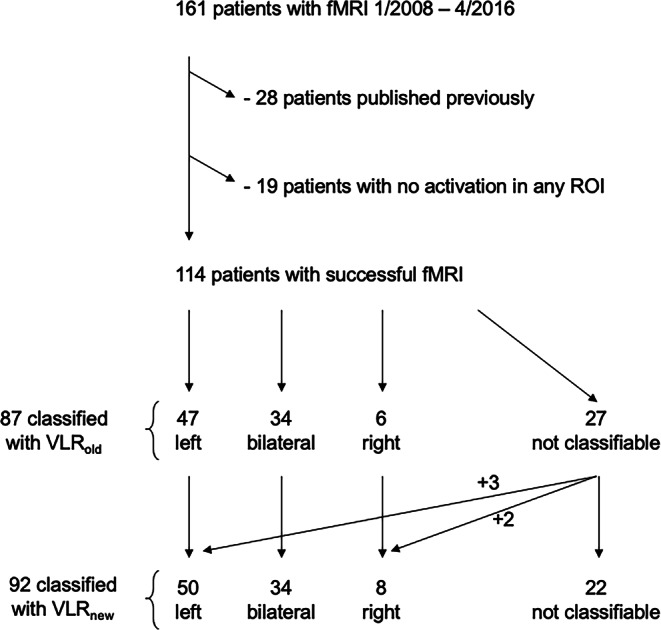
Flowchart of process of patient inclusion and classification of hemispheric language dominance. *ROI* region of interest; *VLR*_*old*_*/VLR*_*new*_ validly lateralizing ROIs from our validation study (VLR_old_ [1]) and from the current study (VLR_new_)

We retrospectively analyzed speech fMRI examinations performed in the center Schön Clinic Vogtareuth during the study period January 2008 to April 2016. Approval by the ethics committee of the Medical Faculty of the University of Tübingen was obtained (reference number: 636/2015BO2). The study cohort comprised 161 children and adolescents younger than 18 years who were examined prior to possible epilepsy surgery. In a first step, 28 patients were excluded whose data had contributed to the preceding validation study [1], as were another 19 patients without activation in any of the typically activating ROIs as defined [1]. Thus, a cohort of 114 patients (58 female, 56 male, mean age 12.5 years; median age 12.7 years; range 5.8–17.8 years) was analyzed (Fig. 1). The native language of the patients varied, with 100 native speakers of German as well as Albanian (*n* = 1), Arabian (*n* = 2), Croatian (*n* = 1), Italian (*n* = 1), Romanian (*n* = 1), Russian (*n* = 4), Slovenian (*n* = 3) and Turkish (*n* = 1) (Supplementary Table 1). Pathologies (radiological or, whenever available, histopathological) comprised focal cortical dysplasia (*n* = 37), benign tumor (*n* = 23), stroke (*n* = 17), mesial temporal sclerosis (*n* = 9), traumatic brain injury (*n* = 5), tuberous sclerosis (*n* = 3), polymicrogyria (*n* = 2), Sturge-Weber syndrome (*n* = 2), mild malformation of cortical development (*n* = 1), autoimmune encephalitis (*n* = 1), brain abscess after sinusitis (*n* = 1), and herpes encephalitis (*n* = 1). Among the 102 patients with identifiable lesions, lesions were located in the left hemisphere in 64 patients, in the right hemisphere in 30 patients, and 8 patients showed bilateral lesions. No clear epileptogenic lesion was identified in 12 patients (Supplementary Table 1).

### FMRI Procedure

Most patients (*n* = 98) underwent only one fMRI examination; in patients undergoing more than one fMRI (*n* = 10 received 2 fMRIs; *n* = 5 received 3 fMRIs; *n* = 1 received 4 fMRIs), only the last examination was analyzed (Supplementary Table 2).

The fMRI scans were performed in a 1.5 T MR scanner (Siemens Symphony, Erlangen, Germany). An echo planar imaging (EPI) sequence was used to obtain functional imaging data (TR = 3000 ms, TE = 40 ms, 40 axial slices of 3 mm thickness, 0.5 mm gap to reduce cross-talk, in-plane matrix = 64 × 64, yielding a voxel size of 3 × 3 × 3 mm^3^), covering the whole brain including the cerebellum. Data analysis was executed by the real-time statistical processing software package of the scanner (syngo® fMRI neuro suite, Siemens inline BOLD imaging). Acquired imaging data were automatically statistically evaluated (Student’s t‑test) to produce BOLD maps. To attain the final fMRI images, BOLD activation maps (color t‑value maps) were superimposed on the averaged EPI images. The software also included retroactive motion correction and smoothing. Standard thresholds were set at T ≥ 4 (activation threshold) and a cluster threshold of 5 voxels, with the need to individually adjust thresholds to adapt to varying degrees of fMRI activation levels in each patient, but at a minimum t‑value of ≥ 3. An overall balance between artifact contamination (false positives) and loss of relevant activation (false negatives) due to overly high thresholds had to be found. The T‑thresholds were adjusted for each session in steps of 0.5 until such a balance was reached.

Children were extensively prepared for the examination. Each task was thoroughly explained and practiced prior to entering the scanner, using original task material [21]. Each patient was equipped with MR-compatible video goggles (Resonance Technology, Los Angeles, CA, USA) for visual stimuli presentation and MR-compatible headphones (Resonance Technology) for auditory task instructions and noise reduction. Furthermore, each participant received one or two controllers (with two buttons per controller). In patients with sufficient function, both hands were used for pressing the response buttons, while in patients with severe hemiparesis, only the non-paretic hand was used for button pressing.

During each fMRI examination, between one and four of the previously established language tasks (VIT, WCT, BST, SYT) were measured in sequential sessions, and most tasks were repeated at least once for reproducibility (Supplementary Table 2). Tasks and their sequential order were individually selected during each examination, depending on patient compliance and the results available during the fMRI procedure. All task sessions were implemented in identical block designs with alternating 30 s blocks of active (linguistic) and control (nonlinguistic) conditions.

For BST and WCT, the same methodology was used throughout the study period. In contrast, for the two decision tasks SYT and VIT, a refined methodology was used in all participants tested after July 2013. Before this time, fixed presentation times (1 stimulus every 5 s) were used [21]. The refined methodology applied after July 2013 comprised a slight modification of the task design through a self-paced component [32], which enables the participants to select the velocity of the slide show themselves, using the presentation software (version 0.76, Neurobehavioral Systems Inc., Albany, CA, USA). With this modification, the rate of successful fMRI sessions was increased especially for patients with high cognitive abilities for whom 5 s per stimulus is too slow and for cognitively impaired patients requiring more than 5 s. The principal activation patterns, however, remained unchanged, so that for the current study, data from these two tasks designs were combined. All examinations were performed in the patients’ first language (Supplementary Table 1). For non-German versions of VIT and SYT, we modified our visual stimuli accordingly. When tasks were performed in more than one language (Supplementary Table 1), the sessions providing more activation were used for analysis.

### Definition and Analysis of Regions of Interest (ROI)

**Fig. 2:**
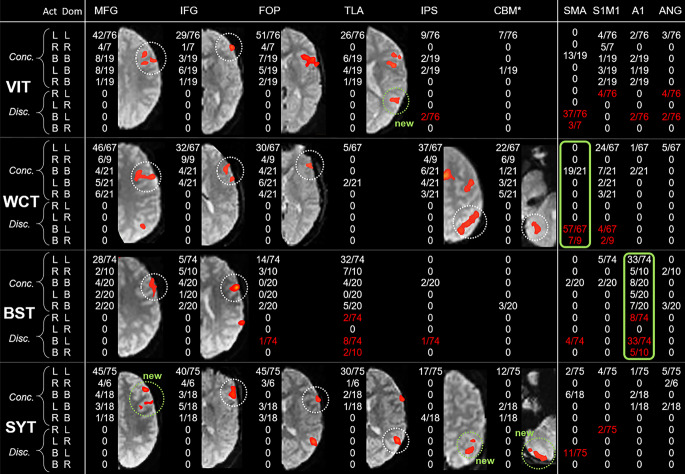
FMRI activation in the 40 task-specific ROIs. For all 4 tasks (*left column*) and all 10 ROIs (*top row*), the proportion of sessions showing concordant (Conc.) and discordant (Disc.) activation (= numerators) on the total number of sessions showing any ROI activation for this task (= denominators) is displayed separately for left-dominant (L), right-dominant (R) and bilateral (B) patients. Discordant activation is marked by *red numbers*. *Act:* *ROI* activation; *Dom* language dominance of the patient (from the subgroup of 69 patients with left, right or bilateral language dominance). Note that bilateral activation (B) was classified as discordant in patients with lateralized language (L or R), while lateralized activation (L or R) was classified as concordant in patients with bilateral language dominance (B). ROIs with no discordant activation and at least 10 activated sessions were classified as validly lateralizing ROIs (VLR) and are visualized by a typical fMRI example (*dotted circles* indicating the respective ROI), overlaid on a mean functional image for anatomical reference. The four additional VLRs identified in the present study are designated as “new” (*green*). Green boxes mark indicator ROIs (see Fig. 5). *Asterisk* For the CBM, right-hemispheric activation was counted as indicating left-dominance and vice versa. *MFG* middle frontal gyrus, *IFG* inferior frontal gyrus, *FOP* frontal operculum, *S1M1* central region, *A1* primary auditory cortex (Heschl’s gyri), *TLA* temporal language area, *IPS* intraparietal sulcus, *ANG* angular gyrus, *CBM* cerebellum, *SMA* supplementary motor area

Based on the typical activation patterns of these tasks nine ROIs were defined using anatomical boundaries as previously described [1, 21, 23, 32]. Each of these ROIs comprised homotopic regions in both hemispheres. Identical to [1], in the frontal lobe activation was analyzed in the middle (MFG) and inferior frontal gyrus (IFG), the frontal operculum (FOP) and the central region (S1M1, from precentral to postcentral sulcus). Temporal ROIs were the primary auditory cortex (A1) (Heschl’s gyri) and the temporal language area (TLA, activation in axial plane posterior to Heschl’s gyri, including the planum temporale, posterior part of superior and middle temporal gyrus). Parietal ROIs were the intraparietal sulcus (IPS) and the angular gyrus (ANG). Furthermore, the cerebellum (CBM) was analyzed. Additionally, the supplementary motor area (SMA) was implemented as a typically activated ROI. Activation of each ROI was evaluated for each of the four language tasks of the fMRI paradigm. Thus, in total 40 task-specific ROIs (4 tasks × 10 ROIs) were included for the present study (Fig. 2). As described above, 13 of these task-specific ROIs had been previously identified as VLRs ([1]; Fig. 2).

### FMRI Processing and Analysis

As clinically implemented, a visual region-of-interest (ROI)-based approach was applied. Analysis was performed visually by an experienced senior pediatric neurologist (MS) for the ten described typically activating ROIs, each comprising homotopic regions in both hemispheres (Fig. 2). Activation was categorized for each session and each of the ten ROIs as “left”/“right”/“bilateral”/“not activated”. “Bilateral” was categorized when the respective ROI showed equally strong activation in both hemispheres, whereas asymmetric bilateral activation was categorized as either “left” or “right”. For comparison with clinical routine examinations, we did not introduce a more quantitative approach to distinguish between unilateral or bilateral, but used this, admittedly subjective, visual assessment. A session was classified as “unsuccessful” when none of these ten ROIs showed any activation or when artefacts made interpretation impossible. This was the case for 156/748 sessions (BST 24/208, WCT 61/196, VIT 37/182, SYT 34/162) (Supplementary Table 2). When all sessions of all tasks were unsuccessful, the patient was excluded as described above (*n* = 19).

### Identification of Language Dominance

We then analyzed hemispheric language dominance of each patient in our cohort using the 13 validly lateralizing ROIs defined in the validation study (VLR_old;_ [1]).

For the current study, we considered a patient safely classifiable when at least three activations of VLR_old_ were observed across all sessions and tasks of the examination. This cut-off was selected in order to obtain sufficiently reliable data in the clinical context. The VLRs showing bilateral activation were counted as two activations (one per hemisphere). To mirror the continuum of language dominance reaching from left dominance to bilateral to right dominance, we introduced a simplified laterality index (LI), which we propose as more applicable for clinical routine than traditionally used calculated voxel-based LIs [33, 34]. For this calculation (Supplementary Table 3), we counted for each hemisphere and across all sessions and tasks of the fMRI examination how often a VLR_old_ was activated, with LI = (sum of left activations − sum of right activations) / (sum of left activations + sum of right activations). As cerebellar language activation occurs in a crossed cerebrocerebellar organization [35–37], right-lateralized cerebellar activation was counted for the left hemisphere and left-lateralized cerebellar activation was counted for the right hemisphere. Aiming to define a subset of left-dominant, right-dominant and equally bilateral patients (Supplementary Table 3), we classified only patients with LI = +1 as “left-dominant”, with LI = −1 as “right-dominant”, and with +0.5 < LI < −0.5 as “bilateral”. Patients in-between these categories, with +1 < LI ≤ +0.5 (“bilateral-left”) or −0.5 ≤ LI < −1 (“bilateral-right”) were omitted during this first step.

### Validly Lateralizing ROI (VLR)

Subsequently, all those patients classified as left, right or bilateral were reanalyzed to validate the 40 task-specific ROIs regarding their ability to lateralize language. In each fMRI session, activation of each ROI was categorized in “concordant” or “discordant” with this classification (Fig. 2). Thus, ROIs showing bilateral activation in patients with left or right dominance were classified as “discordant”. In contrast, for patients with bilateral language, VLR showing lateralized activation were not classified as discordant, allowing that in bilateral patients some ROIs can activate only in the right hemisphere and some only in the left hemisphere in agreement with the concept of crossed dominance [38]. From this reanalysis, a new set of validly lateralizing ROIs (VLR_new_) was identified using two inclusion criteria, namely no discordant activation AND activation in at least 10 sessions. In a next step, we explored in a simulative approach, whether this enlarged set of 17 VLR_new_ would enable us to classify more patients than the old, smaller set of 13 VLR_old._

### Task Order

Finally, to define an optimal order to measure the tasks of our task battery, we then investigated the ability of each task and task combination to identify language. For calculation of these task-specific and/or task combination-specific LIs, not all activated VLR of each patient were used. Instead, we simulated resulting LIs in our 114 fMRI examinations based on the assumption that not all tasks had been measured. Thus, we explored which single tasks and which task combinations would have resulted in the same LI and therefore in the same language lateralization as the complete fMRI examination using all activated VLR for the respective patient.

## Statistics

For statistical calculations quantitative data of the cohort were analyzed using an Excel 2019 software, version 16.51 (Microsoft Corp. Redmond, WA, USA). Qualitative variables were expressed as percentage or points and visualized in Excel 2019-based graphics. Statistical approaches and LI calculation steps can be found in more detail in Supplementary Table 3.

## Results

### Identification of Language Dominance using fMRI

In total, our study material of 114 fMRI examinations comprised 748 task sessions (between 1 and 11 repetitive sessions per fMRI examination) of the 4 different tasks (between 1 and 4 tasks per examination) (Supplementary Table 2). Among the 114 patients in our cohort, 27 showed insufficient (*n* < 3) activation in VLR_old_ and could therefore not be safely classified. The remaining 87 patients were classified as 47 left, 18 bilateral-left, 16 bilateral, 0 bilateral-right, and 6 right (Fig. 1).

### Validly Lateralizing ROI (VLR)

For the purpose of this study, we considered patients as bilateral with a calculated LI of +0.5 < LI < −0.5, indicating activation in both hemispheres in contrast to patients with calculated LI of +1 < LI ≤ +0.5 (“bilateral-left”) or −0.5 ≤ LI < −1 (“bilateral-right”), indicating bilateral language dominance with predominance of one hemisphere, who had been omitted in the first step of analysis. Using only the 69 patients classified as left, right or bilateral and omitting the 18 “bilateral-left” patients with +1 < LI ≤ +0.5, we classified all ROI activations (*n* = 1251) in all sessions into “concordant” (*n* = 906) or “discordant” (*n* = 345) (Fig. 2). At least 1 discordant activation was detected in 14/40 task-specific ROIs (Fig. 2). Of the 26/40 task-specific ROIs with concordant activation exclusively, 17 met the second criterion of activation in at least 10 sessions of the respective task and were therefore identified as “validly lateralizing”. This new set of 17 validly lateralizing ROIs (VLR_new_) (Fig. 2) comprised all 13 validly lateralizing ROIs of the validation study (VLR_old_; [1]) plus four additional validly lateralizing ROIs, i.e., TLA for the VIT, MFG, IPS and CBM for the SYT.

**Fig. 3:**
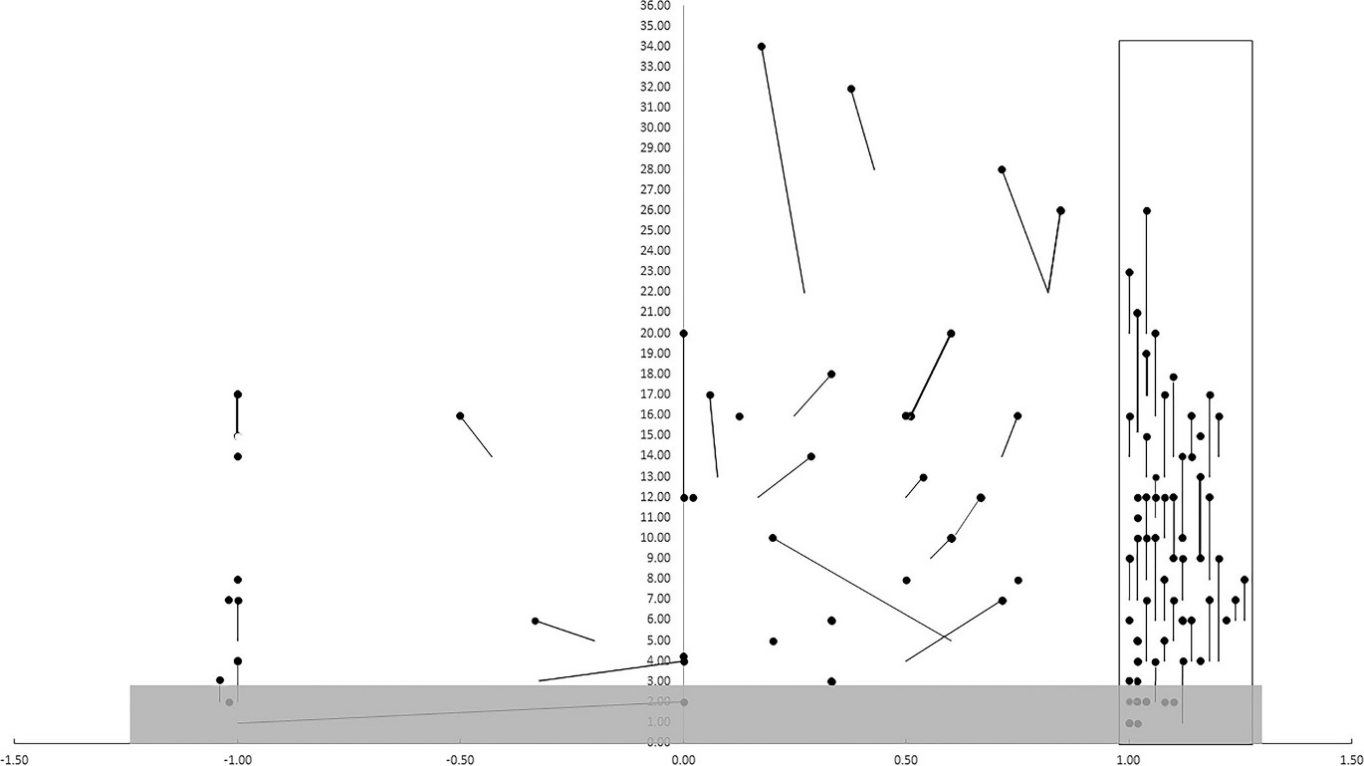
Lateralization indices (LI) calculated based on VLR_old/new_. LIs (x-axis) and number of activated VLR (y-axis) are displayed for all 114 patients. For each patient, a *black dot* represents the number of activated VLR_new_ and the respective LI. When LI/VLR_old_ and LI/VLR_new_ differ, a *black line* links those points. In the 87 patients already classifiable with VLR_old_, minor changes in the degree of bilaterality would be observed in 17 of the 34 patients with any degree of bilateral language (range of LI_old_–LI_new_: from −0.3 to +0.4; median −0.036). The *grey bottom area* marks fMRI examination without sufficient (≥ 3) VLR activation

In a next step, we then simulated application of these 17 VLR_new_ to the initial cohort of 114 patients. If lateralizing activation also of our 4 newly introduced VLR had been used, 92 patients (81%) would have shown a sufficient number (*n* ≥ 3) of activations (Fig. 3). Thus, it would have been possible to classify hemispheric language dominance in 5 more patients (3 left-dominant, 2 right-dominant) than by only applying the 13 VLR_old_ (Fig. 1). This would have increased the success rate (i.e., examinations with at least 3 activations in VLR) of our fMRI examinations from 87/133 (65%) to 92/133 (69%) (Fig. 1).

### Laterality Index (LI)

In the 87 patients already classifiable with VLR_old_, minor changes in the degree of bilaterality would be observed upon simulation with VLR_new_ in 17 of the 34 patients with any degree of bilateral language (range of LI_old_–LI_new_: from −0.3 to +0.4; median −0.036) (Fig. 3; Supplementary Table 3).

### Task Order

**Fig. 4:**
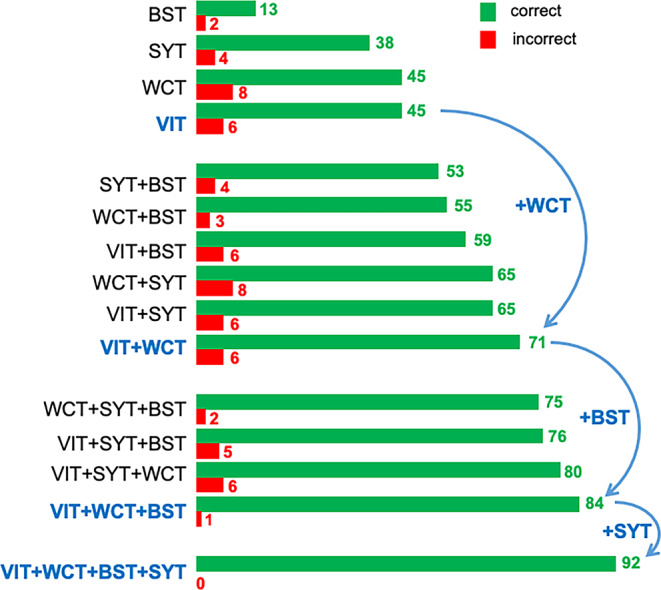
Simulation of language lateralization for reduced number of tasks. The simulated ability of each single task and each possible task combination (*left side*) to identify language dominance in our study population of 92 classified patients is displayed. For each task and task combination, horizontal bars indicate the number of correctly (*green*) and incorrectly (*red*) classified patients. Note that for methodological reasons, an incorrect classification in our sample could only occur when a single task or task combination yielded lateralized results in patients with bilateral language, since all task-specific ROIs with sessions yielding right-lateralized results in left-lateralized patients (and vice versa) had been excluded as discordant. The proposed order to measure the four different tasks is shown with *blue curved arrows*: Start with VIT, then add WCT, then BST, finally SYT. This allows an optimal success rate even when all four tasks cannot be measured, e.g., due to loss of cooperability of the patient in the course of the fMRI examination

Finally, the ability of each task and task combination to identify language was explored. For that aim, we simulated for all 92 patients, which language lateralization would have resulted, if only a reduced number of tasks had been performed (Fig. 4). As single tasks, the VIT and the WCT task showed equally high success rates (45/92 patients correctly classified). False classifications, however, occurred more frequently for WCT (8/34 bilateral patients falsely classified as left-dominant) than for VIT (6/34 bilateral patients falsely classified as left-dominant). The best combination of two tasks was VIT + WCT with a success rate of 71/92 correctly classified patients, albeit still with 6/34 incorrectly classified bilateral patients. The best combination of three tasks was VIT + WCT + BST. Adding BST to VIT + WCT increased the success rate to 84/92 patients and decreased the rate of falsely classified bilateral patients to 1/34. The remaining 8 patients could only be classified by adding SYT to the combination of VIT + WCT + BST.

## Discussion

In the present study, we confirmed all four tasks of our “task battery” [21–23] and all 13 VLR_old_ established in the validation study [1] as useful to determine hemispheric language dominance. Furthermore, we could enlarge this set of 13 VLR_old_ by 4 additional VLRs. Ideally, all four tasks should be measured during one fMRI examination. This might, however, not be possible in all children due to lack of compliance or short attention span. We therefore looked at the success rates and potential pitfalls of all single tasks and task combinations, in order to develop an optimal task order providing reliable information even when the examination has to be stopped before all tasks were measured.

As single tasks, the VIT and the WCT tasks showed equally high success rates (45/92 patients correctly classified). False classifications, however, occurred more frequently for the WCT (8/34 bilateral patients falsely classified as left-dominant) than for the VIT (6/34 bilateral patients falsely classified as left-dominant). We therefore suggest measuring VIT as the first task. This task has the further advantage of being an easy decision task for young children, which allows direct monitoring of task compliance using response buttons [21].

The best combination of two tasks was VIT + WCT, with a success rate of 71/92 correctly classified patients, albeit still with 6/34 incorrectly classified bilateral patients. We therefore suggest measuring WCT as the second task. The WCT, as a silent word generation task, is suitable to monitor the patients’ activity during the task; however, this disadvantage is less important, especially in high-performing patients with expected higher levels of task adherence. In such patients, the VIT might be too easy to induce consistent activation, which explains the increase in success rate by the addition of WCT.

The best combination of three tasks was VIT + WCT + BST. Adding BST to VIT + WCT not only increased the success rate to 84/92 patients but, with the high rate of BST to identify right-hemispheric components of language, the rate of falsely classified bilateral patients dropped to 1/34. Therefore, we suggest measuring the BST as the third task. Whilst the BST was less successful to identify language lateralization as a single task (13/92 patients) it was, being the easiest task, still a valuable tool for younger or cognitively more impaired children who were not able to successfully perform any of the other tasks of our battery. This low success rate is counterbalanced by a comparatively small number of incorrectly classified patients (2/34). Hence, when performing BST as the only possible task, the risk not to obtain any reasonable activation in VLR is higher but the more dangerous risk to obtain false results is lower.

The remaining 8 patients could only be classified by adding SYT to the combination of VIT + WCT + BST, demonstrating that, ideally, all 4 tasks should be measured. Due to its six VLRs and therefore high chance of one of them showing activation, SYT can often provide the additional VLR activation to safely (≥ 3 VLR) classify language in patients with poor activation in the other tasks.

In addition to these strategic findings for measuring and evaluating pediatric speech fMRI, our findings also provide further insights into the neurobiological correlates of activation patterns associated with the tasks of our task battery:

For VIT, we found consistent activation of TLA. This is compatible with Price [39] stating that the planum temporale can be activated during silent speech production in the absence of any auditory input due to its involvement in auditory imagery, working memory and inner speech. Thus, the TLA is associated with auditory motor feedback during overt and silent speech production [39]. Articulation of speech produces sound for the listener that will also be heard by the speaker. Auditory feedback is useful for monitoring and correcting speech errors, especially when speech production is more error prone, e.g., speaking in a second language [39]. Once speech is mastered, auditory feedback is less useful and we do not actively pay attention to the sound of our own voice, which explains why bitemporal activation is less during the self-vocalization of our VIT than during listening to somebody else’s voice in our BST [39]. In contrast, bitemporal activation increases with mismatch between expected and actual auditory feedback, e.g., on a telephone line that delays auditory feedback. The error signal is then fed back to the primary motor cortex to adjust speech output. Similar to this, listening that requires more attention, e.g., to make a decision about an acoustic stimulus [40] or our BST requiring detect and repair mechanisms induce stronger bitemporal activation. This demonstrates that attention enhances auditory cortex activation [40].

Notably, in BST, the FOP, including the classical Broca’s area did not become a validly lateralizing ROI. This is compatible, however, with similar findings among epilepsy patients also showing strong activation of MFG and IFG in the absence of fMRI activation in the traditional Broca’s area [41].

For SYT, MFG, CBM and IPS were introduced as VLR_new_. The SYT is primarily a semantic decision task not merely requiring semantic knowledge of the presented word but rather selection or control strategies to find the correct word with similar meaning. Activation of the MFG is often observed along with inferior frontal activation [24, 31, 39] and has been associated with controlled semantic word retrieval, lexical selection, verbal working memory and phonological processing, playing a mediating part for control, attentional and selection processes [40, 42, 43] as required in SYT to compare the meaning of two words. Activation in the intraparietal sulcus has been attributed to short term memory (verbal and visuospatial) [22, 44, 45] and phonological word processing [46, 47]. The cerebellum is involved in articulation and motor control, but also in higher-order cognition-related components of language [48]. Furthermore, an association with word retrieval has been stated [39], explaining cerebellar activation seen with verbal fluency tasks, word generation tasks like our WCT [1], semantic and phonological processing and phonological short-term memory [49]. The SYT might thus activate the IPS as well as the CBM due to the verbal working memory component of this task, with the need to store the two presented words for comparison and, due to the semantic selection process, to make a decision whether the words are synonyms.

**Fig. 5:**
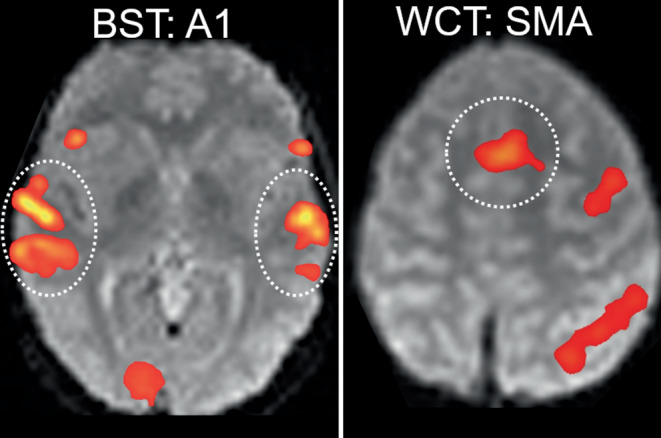
Indicator ROIs. Frequently activated ROIs without lateralizing value can serve as indicator ROIs. This was the case for Heschl’s gyri (A1) (activated in 104/104 sessions, 100%, showing any ROI activation during BST; see Fig. 2) and for the supplementary motor area (SMA) (activated in 83/97 sessions, 86%, showing any ROI activation during WCT; see Fig. 2). These indicator ROIs are visualized by a typical fMRI example (overlaid on the mean functional images); *white circles* mark the respective ROIs

Interestingly, for BST and WCT, the task-specific ROIs showing the most consistent activation were not included in our set of VLR_new_: the primary auditory cortex (A1) for the BST and the supplementary motor area (SMA) for the WCT. These ROIs had been excluded because of “discordance”, and this was mainly due to bilateral activation in left-dominant or right-dominant patients (Fig. 2). This explains, why 141/592 sessions were classified as “successful”, but not lateralizing. Since these ROIs were activated in almost every session that activated VLR_new_, 77/77 (100%) A1 for BST, 103/120 (86%) SMA for WCT, we suggest that activation in these regions can serve as “indicators” for successful fMRI task performance. In other words, we recommend interpreting fMRI activation patterns for BST or WCT with caution when no activation of these “indicator ROIs” (Fig. 5) is present. This approach is compatible with findings of Suarez et al. [50], who also noted that passive story listening induces strong activation of the primary auditory cortex, concluding that absence of this activation indicates that the resulting language map is inconsistent with the language paradigm and therefore not reliable for language lateralization.

Admittedly, our study has several shortcomings. First, our proposed analysis algorithm is based on visual analysis. Thus, our proposed simplified LI obviously cannot aim at achieving the same accuracy as traditionally used voxel calculation-based fMRI-LIs, since it still depends to some extent on the experience of the rater and remains prone to subjective interpretation. Our approach rather aims at standardizing visual interpretation, making it less dependent on rater experience but still applicable in the clinical context, where inferences must be drawn from individual patient’s dataset, even at the cost of suboptimal data quality [51]. This is in accordance with studies showing that visual inspection by an experienced rater is a reliable and valuable method to validate and interpret fMRI in a clinical routine setting [26, 52–59]. Gaillard et al. [54] compared visual assessment of fMRI with quantitative methods such as the LI and found comparable results. Also, Rodin et al. [58] validated qualitative fMRI assessment against LI, reporting even higher concordance rates with the Wada test and with cortical stimulation by visual inspection than with automated analysis algorithms.

Second, the definition of safe classification based on activation of three VLR remains to some extent arbitrary; however, this cut-off was chosen in consideration of clinical fMRIs purpose and over 10 years of clinical experience in visual interpretation of fMRI. For the purpose of language lateralization, not localization, all task-specific VLR activations in the frontal, temporal, parietal lobe and the cerebellum were counted for each hemisphere and across all sessions and tasks of the fMRI examination. Thus, different task-specific VLR might be counted. If considered as a diagnostic “screening- and/or confirmation-tool”, the main purpose of clinical fMRI is not to selectively localize all specific language processing areas but to lateralize hemispheric language dominance and thus identify patients with atypical right or bilateral language representation or to confirm typical left dominance in patients, where this is clinically already expected, like in healthy right-handers, where the left-hemisphere is dominant in up to 95% of persons [4]. In patients with neurologic disorders, such as epilepsy or structural brain lesions, particularly pediatric patients with early onset of disease, atypical language representation is up to approximately 77% higher than in healthy subjects [2–5, 60]. Thus, considering the invasive nature of further diagnostic methods such as the Wada test and electrocortical stimulation (ECS), it is especially helpful to preselect patients with possible atypical language dominance, requiring further invasive testing or identify patients with typically left dominance at risk of language deficits after left-sided surgery, respectively. Even if applying a stricter cut-off of at least 9 VLR_new_ (more than 50% of all 17 VLR_new_), identification of language dominance in 57/114 would still be possible with our protocol. Thus, implemented as a “prescreening-tool”, fMRI would still identify 50% of patients requiring further diagnostics or confirm clinically expected language dominance.

Next, our set of 17 VLR_new_ comprises 11 frontal but only 2 temporal regions. Thus, problems in the identification of patients with a classical interhemispheric dissociation of language (temporal left/frontal right or temporal right/frontal left; [38]) might occur. This could happen with our fMRI protocol when consistent and lateralized activations are only observed in frontal regions, and dissociation is missed simply due to lack of temporal activation; however, this problem seems to be neglectable. First, interhemispheric dissociation is rare (< 1%) [61–63]. Second, when analyzing activation patterns in our 34 patients with bilateral language, we did not identify a single patient who required extrafrontal activations to identify bilaterality (data not shown). In other words, all 34 bilateral patients showed activation of both frontal lobes for at least 1 task.

Finally, the new inclusion criterion of at least ten activated sessions was selected in order to cover a broad spectrum of VLR known from clinical practice to reliably show lateralizing activation in word productive language tasks. Of our four newly introduced VLR, the cerebellum in SYT showed activation in the least number of tasks sessions; however, the lateralizing activation of the cerebellar language areas in its crossed cerebrocerebellar organization has been well established [35–37] and its good concordance with the Wada test already validated for WCT [1].

In conclusion, this study presents an optimized algorithm for measuring and evaluating pediatric language fMRI. We identified a task order to obtain optimal results when not all tasks of our task battery can be measured, and we enlarged our set of validly lateralizing ROIs. Furthermore, we could demonstrate that our task battery can be applied with a high success rate in a clinical pediatric sample and can also identify patients with varying degrees of bilateral language representation.

## Supplementary Information

**Supplementary Table 1** Patient characteristics. *M* male,* f* female,* L* left,* R* right,* T(P)(O)-central* (temporo)-(parietal)-(occipital)-central,* FCD* focal cortical dysplasia,* TS* tuberous sclerosis, *ICB* intracranial bleed,* MCA/PCA* middle/posterior cerebral artery stroke, *MELAS *mitochondrial encephalopathy, lactic acidosis and stroke-like episodes, *AVM *arteriovenous malformation, *ANET *angiocentric neuroepithelial tumour, *DNET* dysembryoplastic neuroepithelial tumors, *mMCD* mild malformations of cortical development, *N/A* not available.

**Supplementary Table 2** Description of performed fMRI examinations. The table lists in detail for each patient the number (*n*) of fMRIs performed, the number (*n*) of tasks used per examination, the total number (*n*) of sessions per task and the number of successful (succ.) task sessions. A session was classified as successful when at least one of the ten ROIs (MFG, IFG, FOP, IPS, S1M1, ANG, A1, TLA, CBM, SMA, compare Fig. 2) showed activation. During each fMRI examination, between one and four of the language tasks (VIT, WCT, BST, SYT) were measured and mostly repeated at least once for reproducibility. *VIT* vowel identification task, *WCT* word-chain task, *BST* beep-story task, *SYT* synonym task.

**Supplementary Table 3** Calculation laterality indices (LI). For each hemisphere VLR activations were counted across all sessions and tasks of the fMRI examination and the LI calculated: LI = (sum of left activations − sum of right activations) / (sum of left activations + sum of right activations). We defined a subset of left-, right-dominant and equally bilateral patients (black frames) with LI = +1 as “left-dominant” (L), with LI = −1 as “right-dominant” (R), and with +0.5 < LI < −0.5 as “bilateral” (BL) and a subset of patients in-between these categories, with +1 < LI ≤ +0.5 (“bilateral-left”, BL L) or −0.5 ≤ LI < −1 (“bilateral-right”, BL R). White rows show patients with < 3 activated VLR, which in our approach were not classifiable (NC). If, in a simulative approach, our newly established VLR VIT-TLA, SYT-MFG, SYT-IPS, SYT-CBM (green), had been implemented, five more patients would show sufficient VLR activation (≥ 3) and could have been classified (green). e.g., for patient number 40 activation was found in FOP in one session of VIT and in FOP for one session of WCT (both VLR_old_). The VLR_new_ TLA in two sessions of VIT would have rendered the additional activation (total of four VLR activations). Thus, language dominance of this patient would become classifiable, when considering VIT-TLA. Note: minor changes in the degree of bilaterality upon stimulation with VLR_new_ are highlighted by red bars. *Lold *sum of left activations (VLR_old_)*, Blold *sum of bilateral activations (VLR_old_), *Rold, *sum of right activations (VLR_old_)*, Lnew* sum of left activations (VLR_new_)*, BLnew *sum of bilateral activations (VLR_new_)*, Rnew, *sum of right activations (VLR_new_)*, LI old laterality index*_*old*_*, LInew laterality index*_*new*_*.*
