# Supplementary material for: Clinical Speech fMRI in Children and Adolescents: Development of an Optimal Protocol and Analysis Algorithm
Source: Clin Neuroradiol. 2021 Oct 6;32(1):185–96. doi: 10.1007/s00062-021-01097-z (PMC8894226; doi:10.1007/s00062-021-01097-z)
Supplement: Supplementary file 1 — Supplementary Table 1 Patient characteristics. M male, f female, L left, R right, T(P)(O)-central (temporo)-(parietal)-(occipital)-central, FCD focal cortical dysplasia, TS tuberous sclerosis, ICB intracranial bleed, MCA/PCA middle/posterior cerebral artery stroke, MELAS mitochondrial encephalopathy, lactic acidosis and stroke-like episodes, AVM arteriovenous malformation, ANET angiocentric neuroepithelial tumour, DNET dysembryoplastic neuroepithelial tumors, mMCD mild malformations of cortical development, N/A not available. [file 62_2021_1097_MOESM1_ESM.pdf]

Patient	Sex	Age at epilepsy	Age at last fMRI	Localization of epilepsy	Etiology	Handedness	native/fMRI language
1	m	4,00	11,92	L frontal	FCD	R	Russian
2	m	10,00	12,17	L frontal	MCA stroke	R	German
3	m	3,00	10,83	L frontal	FCD	R	German
4	m	11,50	16,92	R frontal	cryptogenic	L	Slovenian
5	m	N/A	15,33	multifocal	traumatic brain injury	L	German
6	f	N/A	17,67	L temporo-parietal	FCD	R	German
7	f	12,00	13,17	L fronto-parieto-temporal	ICB of AVM	L	German
8	m	2,83	12,75	L fronto-central	FCD	R	German
9	m	2,00	11,50	L temporal	ganglioglioma	N/A	German
10	m	3,50	16,50	R temporal	FCD	L	German
11	f	1,42	6,08	L temporo-parieto-occipital	FCD	L	Romanian
12	m	3,00	11,67	L fronto-central	FCD	L	German
13	f	10,50	15,67	R centro-parietal	FCD	R	German
14	m	3,83	10,42	R temporal	mesial temporal sclerosis	L	German
15	f	10,00	14,83	L centro-parietal	traumatic brain injury	L	German
16	f	11,00	15,50	L temporal	DNET	R	German
17	m	4,00	16,75	L frontal	pilocytic astrocytoma	R	Turkish/German (bilingual)
18	f	9,00	10,08	L fronto-temporal	ganglioglioma	L	German
19	f	3,50	16,42	L fronto-central	FCD	R	German
20	f	3,00	9,17	L hemispheric	FCD	N/A	German
21	f	15,00	17,75	L fronto-temporal	amygdala tumour	R	German
22	f	12,00	14,83	no focal lesion	cryptogenic	R	German
23	f	7,00	11,33	L temporo-parieto-occipital	FCD	N/A	German
24	f	12,08	15,25	multifocal	traumatic brain injury	N/A	German
25	f	6,83	11,92	L occipital	FCD	N/A	German
26	f	3,00	9,83	L frontal	FCD	R	Slovenian
27	f	14,50	14,83	R temporal	DNET	L	German
28	f	2,50	12,50	left temporal	ganglioglioma	R	Russian
29	f	9,50	12,75	R temporal	mesial temporal sclerosis	R	German
30	m	9,25	9,42	multifocal	traumatic brain injury	N/A	German
31	f	16,67	17,08	R frontal	DNET	R	German
32	f	4,00	16,67	L frontal	FCD	R	German
33	f	4,08	10,08	L occipital	perinatal stroke	N/A	German
34	f	4,00	15,33	L T(P)(O) - central	perinatal stroke	R	German
35	m	N/A	14,42	L temporal	ganglioglioma	N/A	German
36	m	11,50	17,00	L frontal	ganglioglioma	R	German
37	f	12,42	15,83	R T(P)(O) -central	Sturge-Weber-Syndrom	R	German
38	m	5,00	14,50	R temporal	TS	L	German

39	m	4,50	8,33	L parieto-central	cryptogenic	R	German
40	f	3,00	12,58	L temporal	cryptogenic	N/A	Arabian
41	f	4,00	10,67	R central	FCD	L	German
42	f	1,42	10,33	L hemispheric	herpes encephalitis	R/L	German
43	m	10,00	12,00	L frontal	FCD	R	German
44	f	5,50	10,17	L temporal	mesial temporal sclerosis	R	German
45	m	11,00	17,33	L temporal	ganglioglioma	R	German
46	m	5,00	17,00	R temporo-parieto-occipital	FCD	R	German
47	f	6,50	16,58	L occipital	FCD	R	German
48	m	14,00	17,33	left fronto-temporal	FCD	R	German
49	m	4,00	12,58	R fronto-temporal	polymicrogyria	R	German
50	f	11,00	12,67	R parieto-temporal	cryptogenic	L	German
51	f	4,00	13,50	L temporal	mesial temporal sclerosis	N/A	German
52	m	12,00	12,67	R frontal	cryptogenic	N/A	German
53	f	N/A	8,08	L hemispheric	autoimmune encephalitis	L	German
54	f	5,00	15,00	L frontal	FCD	R	German
55	f	4,00	10,58	L temporal	mesial temporal sclerosis	L	German
56	f	1,00	15,67	L temporo-centro-occipital	ganglioglioma	L	Croatian
57	m	6,00	10,33	L temporal	mesial temporal sclerosis	R	German
58	m	5,42	12,17	R temporal	benign tumour	N/A	German
59	m	5,50	14,08	R frontal	polymicrogyria	R	German
60	f	9,00	9,83	L temporal	amygdala tumour	R	German
61	m	15,58	15,67	L fronto-central	ganglioglioma	R	German
62	f	6,92	7,67	R temporal	ANET	R	German
63	m	12,00	16,58	R frontal	FCD	R	German
64	m	2,00	14,67	R temporal	mesial temporal sclerosis	R	German
65	f	6,00	10,67	R hemispheric	mMCD	R	German
66	m	5,00	6,42	L fronto-centro-parietal	Sturge-Weber-Syndrome	R	German
67	m	9,50	15,58	R temporo-parieto-occipital	FCD	R	German
68	m	6,50	13,08	L fronto-central	DNET	R	German
69	m	3,00	13,17	R frontal	FCD	R	German
70	f	2,92	13,58	R temporal anterior and mesial	FCD	L	German
71	m	6,00	11,92	R fronto-centro-parietal	FCD	R	German
72	m	4,50	9,75	L temporo-occipital	DNET	R	German
73	f	2,00	10,17	R frontal	FCD	R	German
74	m	15,92	16,58	L fronto-central	benign tumour	R	German
75	m	16,08	16,33	L frontal	MCA stroke	N/A	German
76	m	9,08	9,42	L centro-parietal	cryptogenic	R	German

77	m	16,42	17,00	L temporo-parieto-occipital	ICB of cavernoma	R	German
78	m	11,17	11,92	R centro-temporo-parietal	perinatal stroke	R	German
79	f	5,00	12,83	R fronto-central	FCD	R	German
80	m	10,50	12,92	R frontal	FCD	L	German
81	f	6,00	13,08	R temporal-(occipital)	FCD	R	German
82	m	1,50	9,08	L temporal	mesial temporal sclerosis	R	German
83	f	3,67	13,75	R frontal	FCD	L	German
84	m	11,25	11,67	L central	ICB of AVM	R	German
85	f	N/A	9,83	L frontal	FCD	N/A	German
86	m	0,50	9,00	multifocal	FCD	R	German
87	f	3,50	7,25	R T(P)(O) -central	FCD	R	German
88	m	2,00	7,58	L parietal	peripartal ICB	L	German
89	m	10,00	11,00	subcortical	pilocytic astrocytoma	N/A	German
90	m	5,25	5,92	L parietal	developmental venous anomaly	N/A	German
91	m	2,00	5,83	L temporal	TS	R	German
92	m	4,00	6,83	R temporal	ganglioglioma	L	German
93	m	0,08	15,83	L temporo-occipital	FCD	L	German
94	f	2,50	12,92	multifocal	TS	R	German
95	f	N/A	16,25	multifocal	MELAS-Syndrom with infarction	N/A	German
96	f	N/A	8,67	L hemispheric	MCA stroke	N/A	German
97	m	6,00	8,08	L temporal	FCD	R	Slovenian
98	m	12,00	13,17	multifocal	MCA stroke	N/A	Arabian (native) /English (foreign)
99	m	2,17	9,58	L fronto-centro-parietal	astrocytoma	R	Russian
100	m	4,08	11,67	L centro-parietal	cryptogenic	R	German
101	f	0,58	8,42	L temporo-parieto-occipital	FCD	L	German
102	f	N/A	14,08	L temporal	cryptogenic	R	German
103	f	4,00	14,08	L parieto-occipital	MCA/PCA stroke	L	German
104	f	9,00	11,50	L fronto-centro-temporo-parietal	cavernous haemangioma	N/A	German
105	f	2,50	7,33	L centro-temporo-parieto-occipital	FCD	R	Albanian
106	f	11,33	13,92	multifocal	traumatic brain injury	N/A	German
107	f	13,00	14,42	L temporo-parieto-occipital	stroke	R	German
108	f	7,00	11,50	L temporal	mesial temporal sclerosis	R	Russian
109	m	14,00	16,33	R frontal	brain abscess after sinusitis	L	German
110	f	3,00	7,50	L centro-parietal	perinatal MCA stroke	L	German
111	m	9,00	10,50	L temporo-parietal	perinatal MCA stroke	L	Italian/German (bilingual)
112	f	0,58	11,83	L fronto-temporal	cryptogenic	R	German
113	f	13,00	14,58	L frontal	cryptogenic	R	German
114	m	0,50	14,42	L temporal	perinatal MCA stroke	L	German
