# Supplementary material for: Clinical Speech fMRI in Children and Adolescents: Development of an Optimal Protocol and Analysis Algorithm
Source: Clin Neuroradiol. 2021 Oct 6;32(1):185–96. doi: 10.1007/s00062-021-01097-z (PMC8894226; doi:10.1007/s00062-021-01097-z)
Supplement: Supplementary file 2 — Supplementary Table 2 Description of performed fMRI examinations. The table lists in detail for each patient the number (n) of fMRIs performed, the number (n) of tasks used per examination, the total number (n) of sessions per task and the number of successful (succ.) task sessions. A session was classified as successful when at least one of the ten ROIs (MFG, IFG, FOP, IPS, S1M1, ANG, A1, TLA, CBM, SMA, compare Fig. 2) showed activation. During each fMRI examination, between one and four of the language tasks (VIT, WCT, BST, SYT) were measured and mostly repeated at least once for reproducibility. VIT vowel identification task, WCT word-chain task, BST beep-story task, SYT synonym task. [file 62_2021_1097_MOESM2_ESM.pdf]

Patient	total fMRIs (n)	total task (n)	total tasks sessions (n)	total tasks sessions succ (n)	total VIT (n)	total VITsucc (n)	total WCT (n)	total WCTsucc (n)	total BST (n)	total BSTsucc (n)	total SYT (n)	total SYTsucc (n)
1	1	2	5	5	0	0	2	2	3	3	0	0
2	1	2	5	4	0	0	3	2	2	2	0	0
3	1	1	2	2	0	0	0	0	2	2	0	0
4	2	1	2	1	0	0	0	0	2	1	0	0
5	1	3	6	5	2	1	0	0	2	2	2	2
6	1	4	9	8	2	2	3	2	2	2	2	2
7	1	2	4	2	2	0	0	0	2	2	0	0
8	1	4	8	6	2	2	2	1	2	1	2	2
9	1	3	6	6	0	0	2	2	2	2	2	2
10	1	4	9	6	2	2	3	2	2	2	2	0
11	1	3	6	6	2	2	2	2	2	2	0	0
12	1	1	3	3	0	0	0	0	3	3	0	0
13	1	4	8	8	2	2	2	2	2	2	2	2
14	3	3	7	6	2	2	0	0	3	2	2	2
15	1	3	6	6	0	0	2	2	2	2	2	2
16	1	3	7	6	2	2	3	3	0	0	2	1
17	1	4	8	8	2	2	2	2	2	2	2	2
18	1	3	4	1	0	0	1	0	1	1	2	0
19	1	1	2	2	0	0	0	0	2	2	0	0
20	1	4	6	3	1	0	2	1	2	2	1	0
21	1	3	6	6	2	2	2	2	0	0	2	2
22	1	3	6	6	2	2	2	2	0	0	2	2
23	1	3	6	5	2	2	0	0	2	2	2	1
24	2	4	8	4	2	2	2	0	2	0	2	2
25	1	3	7	5	0	0	3	2	2	2	2	1
26	1	4	8	8	2	2	2	2	2	2	2	2
27	1	4	8	8	2	2	2	2	2	2	2	2
28	1	3	8	4	0	0	3	3	3	0	2	1

29	1	4	8	5	2	1	2	1	2	2	2	1
30	1	4	6	6	1	1	2	2	1	1	2	2
31	1	4	8	7	2	2	2	1	2	2	2	2
32	2	2	4	3	2	1	0	0	2	2	0	0
33	1	3	6	6	2	2	2	2	2	2	0	0
34	1	3	6	3	2	1	2	0	2	2	0	0
35	1	3	5	4	2	2	1	0	2	2	0	0
36	1	2	6	6	4	4	0	0	2	2	0	0
37	1	2	4	3	2	1	0	0	2	2	0	0
38	1	3	7	4	0	0	3	1	2	2	2	1
39	3	3	7	2	2	1	3	0	2	1	0	0
40	1	4	9	8	2	2	2	2	2	2	3	2
41	1	3	6	5	0	0	2	2	2	2	2	1
42	1	1	2	2	0	0	0	0	2	2	0	0
43	1	3	6	6	0	0	2	2	2	2	2	2
44	1	3	6	6	0	0	2	2	2	2	2	2
45	1	3	6	6	0	0	2	2	2	2	2	2
46	1	2	4	2	2	0	2	2	0	0	0	0
47	1	1	2	2	0	0	0	0	2	2	0	0
48	1	4	7	6	1	1	2	1	2	2	2	2
49	3	1	3	3	3	3	0	0	0	0	0	0
50	1	2	5	5	0	0	3	3	2	2	0	0
51	2	4	9	8	3	2	2	2	2	2	2	2
52	1	3	5	4	2	1	1	1	2	2	0	0
53	1	2	4	4	0	0	2	2	2	2	0	0
54	1	2	4	4	0	0	2	2	2	2	0	0
55	3	3	6	6	2	2	2	2	2	2	0	0
56	2	4	7	6	2	2	1	0	2	2	2	2

57	2	2	6	5	4	3	0	0	2	2	0	0
58	1	1	3	3	0	0	0	0	3	3	0	0
59	1	3	6	6	2	2	2	2	2	2	0	0
60	1	3	6	6	2	2	2	2	2	2	0	0
61	1	1	1	1	0	0	0	0	1	1	0	0
62	1	3	6	6	0	0	2	2	2	2	2	2
63	1	4	8	8	2	2	2	2	2	2	2	2
64	1	4	8	6	2	2	2	1	2	2	2	1
65	1	4	9	7	2	2	3	1	2	2	2	2
66	1	3	6	4	2	0	2	2	2	2	0	0
67	4	4	9	5	3	3	2	0	2	2	2	0
68	1	4	8	8	2	2	2	2	2	2	2	2
69	1	1	3	3	0	0	0	0	3	3	0	0
70	1	2	4	2	0	0	2	2	0	0	2	0
71	1	3	6	6	2	2	2	2	0	0	2	2
72	2	3	6	3	2	1	0	0	2	0	2	2
73	1	2	5	3	0	0	3	2	0	0	2	1
74	1	4	7	7	2	2	2	2	1	1	2	2
75	1	4	9	9	2	2	3	3	2	2	2	2
76	1	4	8	4	2	0	2	0	2	2	2	2
77	2	2	6	4	4	2	0	0	2	2	0	0
78	1	4	8	5	2	2	2	0	2	1	2	2
79	1	4	9	8	2	2	3	2	2	2	2	2
80	1	4	9	7	2	1	3	2	2	2	2	2
81	1	4	8	8	2	2	2	2	2	2	2	2
82	1	3	6	2	2	0	2	0	2	2	0	0
83	1	4	8	7	2	2	2	1	2	2	2	2
84	2	4	9	5	2	1	2	0	2	2	3	2
85	1	3	6	4	2	2	2	0	2	2	0	0

86	1	4	8	8	2	2	2	2	2	2	2	2
87	1	4	8	5	2	2	2	1	2	0	2	2
88	1	4	9	8	2	2	2	2	2	2	3	2
89	1	4	11	2	3	0	3	0	2	2	3	0
90	1	4	8	8	2	2	2	2	2	2	2	2
91	1	4	8	8	2	2	2	2	2	2	2	2
92	1	4	8	8	2	2	2	2	2	2	2	2
93	1	4	8	8	2	2	2	2	2	2	2	2
94	1	4	8	3	2	2	2	0	2	0	2	1
95	1	4	8	4	2	0	2	2	2	2	2	0
96	1	4	8	5	2	1	2	0	2	2	2	2
97	1	4	8	4	2	2	2	0	2	1	2	1
98	1	3	6	4	2	1	0	0	2	1	2	2
99	1	4	8	7	2	1	2	2	2	2	2	2
100	1	4	9	9	3	3	2	2	2	2	2	2
101	1	4	8	3	2	0	2	1	2	2	2	0
102	1	3	5	4	2	2	1	0	0	0	2	2
103	1	2	4	3	2	2	2	1	0	0	0	0
104	1	4	9	6	2	2	2	2	3	0	2	2
105	1	4	8	8	2	2	2	2	2	2	2	2
106	1	4	8	7	2	2	2	2	1	0	3	3
107	1	4	8	7	2	2	2	1	2	2	2	2
108	1	4	10	5	2	1	4	1	2	1	2	2
109	1	4	7	5	2	2	2	2	1	1	2	0
110	2	4	8	8	2	2	2	2	2	2	2	2
111	3	4	8	3	2	1	2	0	2	2	2	0
112	1	4	9	8	2	2	2	2	3	2	2	2
113	1	4	8	6	2	2	2	0	2	2	2	2
114	2	2	4	4	2	2	0	0	0	0	2	2
114	138	361	748	592	182	145	196	135	208	184	162	128
