# Supplementary material for: Clinical Speech fMRI in Children and Adolescents: Development of an Optimal Protocol and Analysis Algorithm
Source: Clin Neuroradiol. 2021 Oct 6;32(1):185–96. doi: 10.1007/s00062-021-01097-z (PMC8894226; doi:10.1007/s00062-021-01097-z)
Supplement: Supplementary file 3 — Supplementary Table 3 Calculation laterality indices (LI). For each hemisphere VLR activations were counted across all sessions and tasks of the fMRI examination and the LI calculated: LI = (sum of left activations − sum of right activations) / (sum of left activations + sum of right activations). We defined a subset of left-, right-dominant and equally bilateral patients (black frames) with LI = +1 as “left-dominant” (L), with LI = −1 as “right-dominant” (R), and with +0.5 < LI < −0.5 as “bilateral” (BL) and a subset of patients in-between these categories, with +1 < LI ≤ +0.5 (“bilateral-left”, BL L) or −0.5 ≤ LI < −1 (“bilateral-right”, BL R). White rows show patients with < 3 activated VLR, which in our approach were not classifiable (NC). If, in a simulative approach, our newly established VLR VIT-TLA, SYT-MFG, SYT-IPS, SYT-CBM (green), had been implemented, five more patients would show sufficient VLR activation (≥ 3) and could have been classified (green). e.g., for patient number 40 activation was found in FOP in one session of VIT and in FOP for one session of WCT (both VLRold). The VLRnew TLA in two sessions of VIT would have rendered the additional activation (total of four VLR activations). Thus, language dominance of this patient would become classifiable, when considering VIT-TLA. Note: minor changes in the degree of bilaterality upon stimulation with VLRnew are highlighted by red bars. Lold sum of left activations (VLRold), Blold sum of bilateral activations (VLRold), Rold, sum of right activations (VLRold), Lnew sum of left activations (VLRnew), BLnew sum of bilateral activations (VLRnew), Rnew, sum of right activations (VLRnew), LI old laterality indexold, LInew laterality indexnew. [file 62_2021_1097_MOESM3_ESM.pdf]

Patient	VIT-MFG			VIT-IFG			VIT-FOP			VIT-TLA			WCT-MFG			WCT-IFG			WCT-FOP			WCT-IPS			WCT-CBM		
	L	BL	R	L	BL	R	L	BL	R	L	BL	R	L	BL	R	L	BL	R	L	BL	R	L	BL	R	L	BL	R
1													2			1			2			1					
2													2														
3																											
4																											
5	1																										
6				2									2			2			2			2			2		
7																											
8	2						2						1						1			1			1		
9													2			2						2			2		
10	2						2			2			2						1								
11							1			2									1								
12																											
13	1						2			2			2			2						2			2		
14	2						2																				
15													2			2						2					
16	1			1					2		2		2			1					3	2					
17							2						2						1			2					
18																											
19																											
20														1		1								1			
21	1	1						2												2						1	1
22	2			2			2			2					2								2				2
23	2			2						2																	
24	2						2																				
25															2	2			2								
26	2			2			2									2			2			2					
27							2																				
28													2			3						3			2		

Patient	BST-MFG			BST-IFG			SYT-MFG			SYT-IFG			SYT-FOP			SYT-TLA			SYT-IPS			SYT-CBM		
	L	BL	R	L	BL	R	L	BL	R	L	BL	R	L	BL	R	L	BL	R	L	BL	R	L	BL	R
1	3																							
2																								
3	2																							
4																								
5						2	1																	
6										2									2					
7																								
8	1						2			2			2			2			2			2		
9										2			2			2						2		
10																								
11																								
12																								
13	2						2																	
14													2											
15			2																					
16															1									
17	1			1						2														
18																								
19																								
20				1																				
21													2											
22										2														
23	2						1			1														
24							2			2			2			2								
25	2			2																				
26	2			2			2			2						2			2			2		
27							2						2						1					
28										1			1											

Patient	VLRold	VLRnew	Lold	Blold	Rold	Llold	Language Dominanc	Lnew	BLnew	Rnew	Llnew	Language Dominance
1	9	9	9	0	0	1	L	9	0	0	1	**L**
2	2	2	2	0	0	1	NC	2	0	0	1	NC
3	2	2	2	0	0	1	NC	2	0	0	1	NC
4	0	0	0	0	0	#DIV/0!	NC	0	0	0	#DIV/0!	NC
5	3	4	1	0	2	-0,3333	BL	2	0	2	0	**BL**
6	14	16	14	0	0	1	L	16	0	0	1	**L**
7	0	0	0	0	0	#DIV/0!	NC	0	0	0	#DIV/0!	NC
8	15	21	15	0	0	1	L	21	0	0	1	**L**
9	14	16	14	0	0	1	L	16	0	0	1	**L**
10	7	9	7	0	0	1	L	9	0	0	1	**L**
11	2	4	2	0	0	1	NC	4	0	0	1	**L**
12	0	0	0	0	0	#DIV/0!	NC	0	0	0	#DIV/0!	NC
13	13	17	13	0	0	1	L	17	0	0	1	**L**
14	6	6	6	0	0	1	L	6	0	0	1	**L**
15	8	8	6	0	2	0,5	BL L	6	0	2	0,5	**BL L**
16	13	17	7	0	6	0,0769	BL	7	2	6	0,0588	**BL**
17	11	11	11	0	0	1	L	11	0	0	1	**L**
18	0	0	0	0	0	#DIV/0!	NC	0	0	0	#DIV/0!	NC
19	0	0	0	0	0	#DIV/0!	NC	0	0	0	#DIV/0!	NC
20	5	5	2	1	1	0,2	BL	2	1	1	0,2	**BL**
21	16	16	3	6	1	0,125	BL	3	6	1	0,125	**BL**
22	16	18	8	2	4	0,25	BL	10	2	4	0,3333	**BL**
23	7	10	7	0	0	1	L	10	0	0	1	**L**
24	10	12	10	0	0	1	L	12	0	0	1	**L**
25	10	10	8	0	2	0,6	BL L	8	0	2	0,6	**BL L**
26	20	26	20	0	0	1	L	26	0	0	1	**L**
27	4	7	4	0	0	1	L	7	0	0	1	**L**
28	12	12	12	0	0	1	L	12	0	0	1	**L**

29	1						1			1			1						1			1						2						1									1									8	10	8	0	0	1	L	10	0	0	1	L
30						1									2		2				1			2			2									1	1						1								1	14	16	2	2	8	-0,4286	BL	2	2	10	-0,5	BL R
31	2									2			1															1						2			2															6	10	6	0	0	1	L	10	0	0	1	L
32																																																				0	0	0	0	0	#DIV/0!	NC	0	0	0	#DIV/0!	NC
33							2			1			1	1					2				1																													9	10	5	2	0	0,5556	BL L	6	2	0	0,6	BL L
34	1						1																																													2	2	2	0	0	1	NC	2	0	0	1	NC
35				2																																																2	2	2	0	0	1	NC	2	0	0	1	NC
36	1	1								3																					1																					4	7	2	1	0	0,5	BL L	5	1	0	0,7143	BL L
37							1																																													1	1	1	0	0	1	NC	1	0	0	1	NC
38															1															1						1																2	3	0	0	2	-1	NC	0	0	3	-1	R
39																																																				0	0	0	0	0	#DIV/0!	NC	0	0	0	#DIV/0!	NC
40	2			2			2				2		1	1			2		2				2					1	1			2					2															28	32	12	8	0	0,4286	BL	12	10	0	0,375	BL
41													1	1					2			1			1				2					1						1												12	13	6	3	0	0,5	BL L	7	3	0	0,5385	BL L
42																																																				0	0	0	0	0	#DIV/0!	NC	0	0	0	#DIV/0!	NC
43																												2						1						2												4	5	4	0	0	1	L	5	0	0	1	L
44																			2			2			2				2					2																		10	12	6	2	0	0,6	BL L	8	2	0	0,6667	BL L
45													2									2			2			2												2			2									12	12	12	0	0	1	L	12	0	0	1	L
46														2						2			2																													12	12	0	6	0	0	BL	0	6	0	0	BL
47																																																				0	0	0	0	0	#DIV/0!	NC	0	0	0	#DIV/0!	NC
48	1										1											1								1					1	1							2									5	10	4	0	1	0,6	BL L	4	2	2	0,2	BL
49	1									3																																										1	4	1	0	0	1	NC	4	0	0	1	L
50																		3						2			2																									4	7	0	0	7	-1	R	0	0	7	-1	R
51				2			2						1			1						1						2			2						2			2			2			2						17	19	17	0	0	1	L	19	0	0	1	L
52			1							1																																										1	2	0	0	1	-1	NC	1	0	1	0	NC
53																						2							2																							6	6	2	2	0	0,3333	BL	2	2	0	0,3333	BL
54													2			2						2											1																			7	7	6	0	1	0,7143	BL L	6	0	1	0,7143	BL L
55	2						2							2		2			2				2																													16	16	8	4	0	0,5	BL L	8	4	0	0,5	BL L
56							2																								2						2			2				2								12	12	8	2	0	0,6667	BL L	8	2	0	0,6667	BL L
57	3																																																			3	3	3	0	0	1	L	3	0	0	1	L

58																																	2																			2	2	0	0	2	-1	NC	0	0	2	-1	NC
59	1			1									1			1												1																								5	5	5	0	0	1	L	5	0	0	1	**L**
60	1			1						2						1						2																														5	7	5	0	0	1	L	7	0	0	1	**L**
61																															1																					1	1	1	0	0	1	NC	1	0	0	1	NC
62																2			2									2												2			2						2			10	12	10	0	0	1	L	12	0	0	1	**L**
63			2												2			2			2			2			2															2										12	14	0	0	14	-1	R	0	0	14	-1	**R**
64		1		2			2							1		1			1																		1			1												12	12	8	2	0	0,6667	BL L	8	2	0	0,6667	**BL L**
65	2						2						1									1			1			2						2						2												11	13	11	0	0	1	L	13	0	0	1	**L**
66																																																				0	0	0	0	0	#DIV/0!	NC	0	0	0	#DIV/0!	NC
67									3																								1																			4	4	0	0	4	-1	R	0	0	4	-1	**R**
68	2												2						2															2																		6	8	6	0	0	1	L	8	0	0	1	**L**
69																																																				0	0	0	0	0	#DIV/0!	NC	0	0	0	#DIV/0!	NC
70															2			2			2						2																									6	8	0	0	8	-1	R	0	0	8	-1	**R**
71	1			2			2						2			2			2			1												2			2			2						2						16	20	16	0	0	1	L	20	0	0	1	**L**
72																																		2												1						0	3	0	0	0	#DIV/0!	NC	3	0	0	1	**L**
73													1			1	1					1			2												1															8	8	6	1	0	0,75	BL L	6	1	0	0,75	**BL L**
74	2						2						2						2			2			2															1						1			1			13	15	13	0	0	1	L	15	0	0	1	**L**
75							1			2						3			3			3												2						1			2									13	17	13	0	0	1	L	17	0	0	1	**L**
76																																		2			2			2												4	6	4	0	0	1	L	6	0	0	1	**L**
77	2			2			2																																													6	6	6	0	0	1	L	6	0	0	1	**L**
78		2			2																																	2														12	12	0	6	0	0	BL	0	6	0	0	**BL**
79			2	2									2			2						2									2						2															14	14	12	0	2	0,7143	BL L	12	0	2	0,7143	**BL L**
80	1			1									2			2						2			2									2			2			2						2						14	18	14	0	0	1	L	18	0	0	1	**L**
81	2						2			2				2					2				2								2			2																		16	20	8	4	0	0,5	BL L	12	4	0	0,6	**BL L**
82																												1	1																							3	3	1	1	0	0,3333	BL	1	1	0	0,3333	**BL**
83				1			1																					1						1			2			2			2						2			9	12	9	0	0	1	L	12	0	0	1	**L**
84			1																														2			2			2													5	7	0	0	5	-1	R	0	0	7	-1	**R**
85			1		1			1				1																																								5	6	0	2	1	-0,2	BL	0	2	2	-0,3333	**BL**

86		2						2			2		2			2			2						2										2								2		2				2	2			22	34	8	6	2	0,2727	BL	10	10	4	0,1765	BL
87		2						2		2															1										2			1	1									2				12	20	1	5	1	0	BL	3	7	3	0	BL
88	2						2			2			2			2																		2						2												10	14	10	0	0	1	L	14	0	0	1	L
89																																																				0	0	0	0	0	#DIV/0!	NC	0	0	0	#DIV/0!	NC
90		1					1								2				2													2		2						1												12	14	4	3	2	0,1667	BL	6	3	2	0,2857	BL
91							2						2			2			2			2			2									2			2															14	16	14	0	0	1	L	16	0	0	1	L
92							2			2																														2												4	6	4	0	0	1	L	6	0	0	1	L
93								2				2	2			2			2			2			2			2			2			2			2			2						2						22	28	18	2	0	0,8182	BL L	22	2	2	0,7143	BL L
94				2			2																											1			1			1			1			1						7	9	7	0	0	1	L	9	0	0	1	L
95																2						2			2							2																				10	10	6	2	0	0,6	BL L	6	2	0	0,6	BL L
96							1																																	1												2	2	2	0	0	1	NC	2	0	0	1	NC
97																																																				0	0	0	0	0	#DIV/0!	NC	0	0	0	#DIV/0!	NC
98										1																								2			2						2									4	7	4	0	0	1	L	7	0	0	1	L
99			1			1			1						2			2												2			2			2			2			1			1							13	17	0	0	15	-1	R	0	0	17	-1	R
100	3						3						2																					2						2												10	12	10	0	0	1	L	12	0	0	1	L
101													1									1						2																								4	4	4	0	0	1	L	4	0	0	1	L
102	2						2			2																								2						2			2									8	12	8	0	0	1	L	12	0	0	1	L
103			1									2			1																																					2	4	0	0	2	-1	NC	0	0	4	-1	R
104	2			2			2			1			2			2			2			2			2									2			2			2												20	23	20	0	0	1	L	23	0	0	1	L
105				2			2			2																																	2									6	8	6	0	0	1	L	8	0	0	1	L
106				2			2						2			2			2						2												3															15	15	15	0	0	1	L	15	0	0	1	L
107				2															1			1																		2												6	6	6	0	0	1	L	6	0	0	1	L
108													1																								2			1			2						1			6	7	6	0	0	1	L	7	0	0	1	L
109							2						2						2																																	6	6	6	0	0	1	L	6	0	0	1	L
110	2			2			2						2			2						2			2							2		2			2			2						2						22	26	18	2	0	0,8182	BL L	22	2	0	0,8462	BL L
111	1			1																										2																						4	4	2	0	2	0	BL	2	0	2	0	BL
112	2			2						2			2									1												2			2															9	13	9	0	0	1	L	13	0	0	1	L
113	2			2			2			2																			2											2			2									14	16	10	2	0	0,7143	BL L	12	2	0	0,75	BL L
114				1			1			1																								2									2			2						4	9	4	0	0	1	L	9	0	0	1	L
